# The Use of Physical Restraint in Norwegian Adult Psychiatric Hospitals

**DOI:** 10.1155/2015/347246

**Published:** 2015-11-23

**Authors:** Rolf Wynn

**Affiliations:** ^1^Department of Clinical Medicine, Faculty of Health Sciences, UiT The Arctic University of Norway, 9037 Tromsø, Norway; ^2^Department of Psychogeriatric Services, Division of Mental Health and Addictions, University Hospital of North Norway, 9291 Tromsø, Norway

## Abstract

*Background.* The use of coercion within the psychiatric services is problematic and raises a range of ethical, legal, and clinical questions. “Physical restraint” is an emergency procedure used in psychiatric hospitals to control patients that pose an imminent physical danger. We wished to review the literature published in scientific peer-reviewed journals describing studies on the use of physical restraint in Norway, in order to identify the current state of knowledge and directions for future research.* Design*. The databases PubMed, PsycINFO, CINAHL, Web of Science, and Embase were searched for studies relating to physical restraint (including holding) in Norwegian psychiatric hospitals, supplemented with hand searches.* Results*. 28 studies were included. Most of the studies were on rates of restraint, but there were also some studies on perceptions of patients and staff, case studies, and ethnographic studies. There was only one intervention study. There are differences in use between wards and institutions, which in part may be explained by differences in patient populations. Staff appear to be less negative to the use of restraint than patients.* Conclusions*. The studies that were identified were primarily concerned with rates of use and with patients' and staff's perspectives. More interventional studies are needed to move the field forward.

## 1. Introduction

The use of coercion within the psychiatric services is problematic and raises a range of ethical, legal, and clinical questions [1, 2]. Physical restraint, also referred to as “mechanical restraint,” is a technique whereby a patient is physically restrained so that the range of movement is restricted. Typically, the patient is restrained to a bed, with a belt over the chest area and four belts restraining each limb (“five-point fixation”) [3–5]. Other types of physical restraints, such as walking restraints and special clothing, are rarely used in Norway. Staff that physically hold a patient against the patient's will might also be considered as carrying out a type of physical restraint (“holding”).

In Norway, physical restraint (including holding) may be used as an emergency intervention, with the purpose of increasing the safety of the patient in question and/or fellow patients and staff or avoiding significant damage to buildings and objects [6, 7]. The intervention is typically used when patients are violent or self-harming [3, 8, 9]. It is in Norway not permitted to use physical restraint for therapeutic purposes (i.e., “behavioural treatment”) or as punishment, and the use of physical restraint within the psychiatric hospitals is strictly regulated and monitored [6].

The use of physical restraint is challenging from both a clinical viewpoint and an ethical viewpoint. Subjecting patients to physical restraint carries a risk of physical and psychological harm to patients; staff may be physically harmed when restraining patients, and it may harm the provider-patient relationship and further treatment [10, 11]. Moreover, it is a goal to base psychiatric treatment—to the extent possible—on voluntary cooperation and to subject patients to as little coercion as possible [6].

In recent years, there has in Europe and the US, as well as in other parts of the world, been an increasing focus on the need to reduce coercion within the psychiatric services [2, 11], and more research has been carried out in this area. The purpose of this review was to search and appraise peer-reviewed scientific publications documenting the use of physical restraint in Norwegian hospital-based adult psychiatric services.

## 2. Material and Methods

### 2.1. Strategy for Database Search

**Figure 1:**
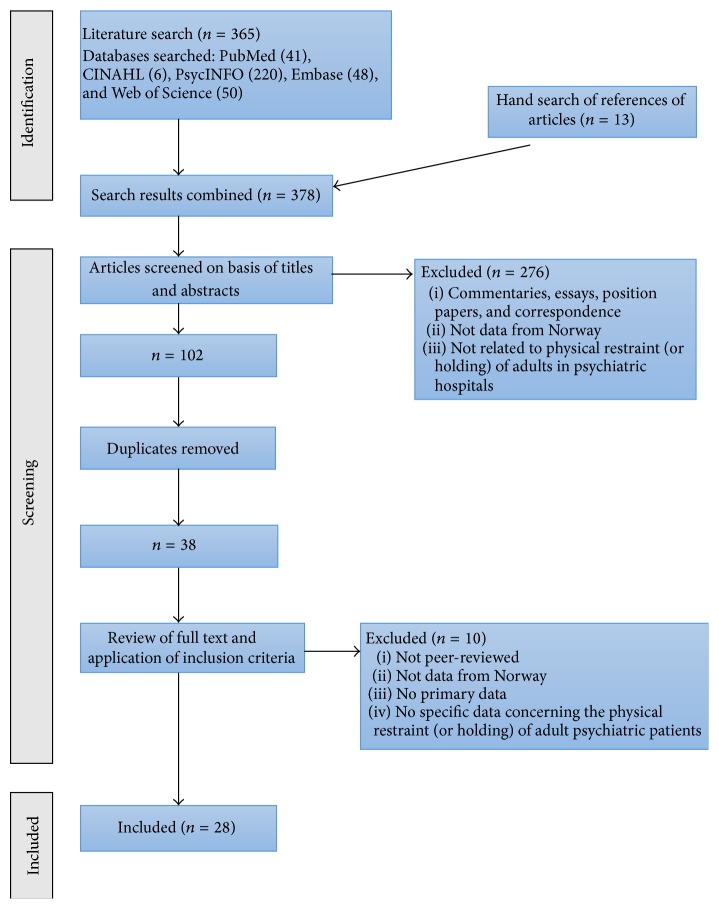
Process for selection of studies included in the review.

A literature search of relevant electronic databases, including PubMed, PsycINFO (Ovid), Embase (Ovid), Web of Science, and CINAHL (EBSCO), was performed to identify relevant articles. The search was carried out in September 2015. The search terms used were various combinations of psychiatry, Norway, physical restraint, mechanical restraint, and holding. The search terms were in English, as Norwegian scientists typically publish in English to reach a wider audience. There are a very small number of Norwegian scientific peer-reviewed journals that might publish relevant articles in the Norwegian language, such as the Journal of the Norwegian Medical Association. However, these journals typically provide English abstracts that are indexed in one or more of the databases that were searched. Hand searches were done of the reference lists of those articles that were identified as relevant to the study. The details of the search process can be found in Figure 1.

### 2.2. Inclusion and Exclusion of Studies

A total of 365 articles were identified in the initial electronic search. Hand searching of the references of articles gave an additional 13 potentially relevant articles. The titles and abstracts of the publications were screened for relevance, reducing the number to 102. When duplicates were removed, 38 articles remained, and these were read in full and considered against the inclusion and exclusion criteria (see below). This gave the final 28 articles that were included in the review (see Table 1 and Figure 1).

### 2.3. Inclusion and Exclusion Criteria

All articles published in peer-reviewed journals carried out with data from Norway and involving the use of physical restraint (including physical holding by staff) within adult psychiatric hospitals were considered for inclusion. Articles not specifically mentioning restraint or that examined coercion only as a general concept were excluded. Studies only involving municipal services, nursing homes, and services for the intellectually disabled were excluded. Studies without data from Norway were excluded, as were studies that did not include primary empirical data (i.e., literature reviews, etc.). Surveys on rates of use of restraint in Norway that had not been published in peer-reviewed journals were not included in the review, but some have nevertheless been mentioned in Discussion.

### 2.4. Data Synthesis and Analysis

The 28 included articles were grouped into five different categories according to their study purposes and methods used. The key findings in each article in the different categories were condensed and the common findings in the various categories were identified.

## 3. Results

The 28 articles were categorized according to their study purposes and methods used (see Table 1). 27 articles described observational studies and only one article [31] described an interventional study.

21 of the studies followed a quantitative methodology. Seven articles had a qualitative design, that is, in-depth interviews [13], case studies [32–34], and ethnographic designs [35–37].

The following categorization was devised: (1) studies examining patients' perceptions of physical restraint, (2) studies examining staff's perceptions of physical restraint by means of questionnaires, (3) studies examining the rates of use of physical restraint and factors that influence such rates; these studies were typically reviews of protocols and/or medical records at one or more departments at one or more hospitals, (4) studies examining the effects of interventions on reducing physical restraint, (5) case studies of individual patients subjected to physical restraint, and (6) ethnographic studies.

### 3.1. Studies Examining Patients' Perceptions of Physical Restraint

Three studies were identified [12–14] that treated the topic of patients' perceptions of physical restraint. One study used only questionnaires filled in by patients [12]; one study used questionnaires filled in partly by patients and partly by researchers as well as data from medical records [14], while one study used in-depth interviews with patients [13].

#### 3.1.1. Main Findings

In one study [12], 19 patients subjected to coercive interventions were matched to controls. There was no significant difference in satisfaction between coerced and matched noncoerced patients; however only three of the included patients had been subjected to physical restraint. An interview study of 12 patients following physical restraint [13] found that patients gave refusal of medication, refusal to follow staff directions, or their own aggression as reasons for restraint. Many felt that restraint could have been avoided, and some felt angry and distrustful of staff after restraint. Iversen et al. [14], drawing on data from 173 patients, found that objective coercion (including mechanical restraint) had a significant negative effect on overall patient satisfaction.

### 3.2. Studies Examining Staff's Perceptions of Physical Restraint by means of Questionnaires

Four studies [15–18] examined the topic of staff's perceptions of physical restraint by means of questionnaires. Two studies used a traditional questionnaire format [15, 16], while two studies used simulated cases, where staff responded to vignettes describing typical situations where restraint was believed to be an option [17, 18].

#### 3.2.1. Main Findings

A study based on questionnaires from 85 staff at one psychiatric hospital found that patients' assault, acting-out, and self-harming were given as most important reasons for physical restraint. 80% of staff believed that physical restraint was used appropriately, and 94% believed that it did not influence patients' recovery [15]. In a study involving questionnaires to 267 staff at a hospital, a majority of staff believed that the interventions were used correctly. Male staff, highly educated staff, and staff at high-use wards were most critical to use. 70% had been assaulted in connection with the interventions [16]. The same questionnaire also included simulated cases, and staff preferred informal interventions above physical restraint [17]. Such informal interventions were typically not recorded and their importance may therefore have been overlooked. A study involving 180 staff at two adult psychiatric units found that there was a limited degree of variance in staff's responses with respect to degree of restrictiveness. The study supported the idea that a range of different interventions are used in emergency situations [18].

### 3.3. Studies Examining Rates of Use of Physical Restraint and Factors That Influence Such Rates

These studies were typically reviews of protocols and/or medical records at one or more departments at one or more hospitals. With one exception [9], all the studies in this category had a retrospective design.

#### 3.3.1. Main Findings

One study from 1983 of a forensic psychiatric hospital found that the use of restraint had been reduced dramatically from more than 15,000 patient days in 1977 to only 1.5 patient days in the first third of 1981 [19]. Høyer and Drange [20], in an examination of protocols of restraint, found that, during the first six months of 1988, 203 patients had been mechanically restrained for 10,767 hours. In a similar study in 1994 [21], they found that 9402 hours of mechanical restraint was recorded in the first half of 1990. Single patients created large variations in use. There was no correlation between size of ward or staff ratio and use of coercion and no difference in levels from the previous study.

Linaker et al. [22], examining the use of restraint in Norwegian security units, found that 25% had been subjected to physical restraint during a six-month period in 1993. Wynn [23], reviewing protocols and medical records from a psychiatric hospital during a 5.5-year period, found that there was a daily peak with most use of restraint in the afternoon and early evening and a seasonal peak—with the most use of restraint in autumn. Patterns of use might be caused by light-dark cycles, variations in life events, and variations in the ward environment. A different article from the same study [24] also demonstrated that there had been 797 episodes of physical restraint and that male, younger, and nonpsychotic patients more often were subjected to physical restraint.

Knutzen et al. [25], in a retrospective study of routinely collected data at one emergency psychiatric department, found that 14% of the patients were subjected to physical and/or pharmacological restraint. The rate of restraint was significantly higher among patients with immigrant background, especially in the younger age groups. Langsrud et al. [26] found that holding the patient with force was more frequent in those incidents where more than one body part had been injured. Husum et al. [9], in a study of 1014 involuntarily admitted patients, found that 117 had been physically restrained and that there was an increased risk of restraint for patients that were aggressive or had a tendency to self-harm. There was a lower risk for patients from other ethnic groups, but an increased risk in urban areas. In a case-control study of patients in three acute wards, Knutzen et al. [27] found that those who had been restrained (mechanically and pharmacologically) were more likely to be male, reside outside the catchment area, have an immigrant background, have longer stays, be involuntarily admitted, and have specific diagnoses (substance use, schizophrenia, psychoses, and bipolar disorder).

In a study of records of 306 patients who had been subjected to physical restraint, Knutzen et al. [8] found that occurring or imminent assault was the most frequent reason for restraint. Moreover, they found that diagnoses, age, and reason for restraint increased the likelihood of being subjected to specific types of restraint, that is, physical or pharmacological. Knutzen et al. [28], in a retrospective study of records data from three acute wards, found that 9.1% of those restrained (either mechanically or pharmacologically) had been so 6 or more times, accounting for 39.2% of all restraint episodes. In a cross-sectional survey of psychiatric units in Norway and Denmark, Bak et al. [29] found that mandatory review, patient involvement, and no crowding were preventive factors. Bak et al. [30] found that the following factors were found to partly explain differences in restraint levels: staff education, substitute staff, acceptable work environment, separation of acutely disturbed patients, patient-staff ratio, and the identification of the patients' crises triggers.

### 3.4. Studies Examining the Effects of Various Interventions on Reducing Rates of Physical Restraint

The only intervention study identified was by Sørgaard, who used three interventions: patients' engagement in the formulation of treatment plans, patient and staff evaluations, and renegotiations of treatment plans [31].

#### 3.4.1. Main Findings

Sørgaard [31] found that the interventions resulted in marginal changes (i.e., in the staff's respect and understanding and total satisfaction). Actions taken to control behaviour (i.e., seclusion) were more strongly related to perceived coercion than aspects of compulsory treatment.

### 3.5. Case Studies of Individual Patients Subjected to Physical Restraint

Three case studies were identified [32–34]. These studies combined literature reviews of the topics discussed with presentation and discussion of cases involving patients that had been subjected or attempted subjected to physical restraint.

#### 3.5.1. Main Findings

All of the studies warned against physical complications that may arise as a consequence of attempting to restrain a patient or actually restraining a patient. Hem and coworkers [32, 33] found that immobilization and trauma to the legs while restraining a patient may lead to thrombosis. Nissen et al. [34] found that physically restraining (holding) a patient in the prone position with a significant weight load on the torso can lead to asphyxiation.

### 3.6. Ethnographic Studies

Three studies had an ethnographic approach. One involved observation and interviews with five patients and six nurses of an open seclusion unit [35], while two other articles [36, 37] were from the same study involving 12 patients and 22 professionals.

#### 3.6.1. Main Findings

A central finding in the study by Hem et al. [35] was that distrust is prevalent, but trust can be created. Regarding themselves as potential causes of distrust can contribute to nurses' developing a realistic view of their practice. Larsen and Terkelsen [36] described the negative experiences of one patient subjected to physical restraint. Terkelsen and Larsen [37] described how physical restraint could be seen as a threat, as a means of control, and as a reminder of potential danger.

## 4. Discussion

A main finding of this review was that half of the included articles (14 of 28) were on rates and factors that influenced rates, especially patient-related factors, such as patients' sex, age, ethnicity, diagnoses, level of aggression, legal status, and duration of stay. A few studies described the importance of organizational or staff-related factors on rates of restraint, including staff-patient ratios, ward size, and staff education levels. Despite this being the category with the highest number of articles, the differences in samples, study designs, and outcomes make it challenging to aggregate the findings. Methodological challenges of this kind have also been highlighted by researchers assessing data from other countries [38–40].

In the present review, one study [25] found that 14% of the patients at an emergency ward had been physically and/or pharmacologically restrained, while another study [9] found that 10% of the involuntarily admitted patients had been physically restrained. These figures are higher than the aggregated numbers reported in the most recent national surveys (2012) [41], where 4.6% were reported to have been physically restrained (and 4.9% had been held). National surveys have also suggested that there are substantial differences in rates between different institutions and possibly also in how different hospitals register and report the use of physical restraint [42]. Routine reporting of physical restraint may be incomplete [43]. However, differences in patient populations may explain some of the differences found in use, with more use in emergency and forensic wards, with involuntarily admitted patients, and with patients with certain diagnoses [3, 27, 44]. Two of the older studies included, examining forensic populations [19, 22], found even higher rates of physical restraint. From 2007, the regulations were revised and holding was also subject to registration. These and other changes in regulations make it difficult to compare older and newer studies on the use of restraint. While one older study included in the review [19] described a dramatic reduction in use of restraint at a single institution at the beginning of the 1980s, studies and surveys with data from the late 1980s and the 1990s showed a lower and more stable use [20, 21, 45]. Several more recent national surveys have found that rates have remained relatively stable [38, 46] and comparable to the rates in other Western European countries [47].

Four studies were concerned with staff perceptions. Overall, the studies suggested that staff believed that restraint was carried out correctly, although there were some differences in opinion between different groups of staff. There were also some differences in opinion as to when physical restraint should be used. Three studies dealt with patients' perceptions. The studies suggested that the patients felt that restraint could have been avoided and most patients reacted negatively to the experience. There were also three ethnographic studies, in part describing staff and patient perceptions, underlining the negative experience of patients to this intervention. Three articles presented cases discussed in the relevant literature, emphasizing physical complications that may arise in connection with the use of restraints or holding [10].

A main finding in this review was that nearly all the identified studies on restraint in Norwegian adult psychiatric hospitals were observational, with only one exception. While there have been several interventional projects with the aim to reduce restraint, these appear not to have been published in peer-reviewed and indexed journals. A next step in the development of the research in this field of research in Norway would be increasing the number of interventional studies and ensuring that they follow needed scientific rigour in order to allow their publication in scientific journals. Researchers in other countries have also pointed to the need to increase the number of high quality intervention studies in this field [48, 49], and some such studies have been carried out in other countries [48, 50, 51]. Studies examining the effects of early identification protocols, deescalation protocols/teams, implementation of the least restrictive alternative model, or systematic debriefing [5, 50] would be examples that would contribute substantially to the field.

The present review showed that there was some variation in the quality of the identified studies, and intervention studies were almost nonexistent. While it might be challenging to carry out intervention studies in this field [2], there is in Norway as in many other countries a need for more studies with a design that will further increase the quality of the evidence and move the field forward [48–51].

The number of studies used in the present review is relatively small as the number of studies in this field is still quite limited. While it is likely that the search captured nearly all the relevant studies that have been published in peer-reviewed journals, there is a possibility that some studies published in journals not indexed by the databases might have been missed.

## 5. Conclusions

The studies that were identified were primarily concerned with patients' and staff's perspectives and rates of use. There is a need for interventional studies with stronger designs.

## Figures and Tables

**Table 1:** Included articles (*n* = 28).

Number	Category	Articles [reference]	Methods/participants (*n*)	Key findings
1	Patients' perceptions (1)	Patients' satisfaction and self-rated improvement following coercive interventions [12]	Questionnaire to adult psychiatric patients (total sample *n* = 410, of which 19 subjected to coercion were matched to other patients) (only 3 had been physically restrained)	No significant difference in satisfaction between coerced and matched noncoerced patients

2	Patients' perceptions (1)	Psychiatric inpatients' experiences with restraint [13]	Interviews with adult psychiatric patients (*n* = 12) following physical restraint	(i) Patients gave refusal of medication, refusal to follow staff directions, or their own aggression as reasons for restraint (ii) Many felt that restraint could have been avoided (iii) Some felt angry and distrustful of staff after restraint

3	Patients' perceptions (1)	Coercion and patient satisfaction on psychiatric acute wards [14]	173 interviews and questionnaires with patients, data from medical records	Objective coercion (including mechanical restraint) had a significant negative effect on overall patient satisfaction

4	Staff's perceptions (2)	Staff's experiences with patients' assaults in a Norwegian psychiatric university hospital [15]	Questionnaire to staff at one adult psychiatric hospital (*n* = 85)	(i) Patients' assault, acting-out, and self-harming were given as most important reasons for physical restraint (ii) 80% of staff believed that physical restraint was used appropriately, and 94% believed it did not influence patients' recovery

5	Staff's perceptions (2)	Staff's attitudes to the use of restraint and seclusion in a Norwegian university psychiatric hospital [16]	Questionnaire to staff at one adult psychiatric hospital (*n* = 267)	(i) A majority of staff believed that the interventions were used correctly (ii) Male staff, highly educated staff, and staff at high-use wards were most critical to use (iii) 70% had been assaulted in connection with the interventions

6	Staff's perceptions (2)	Staff's choice of formal and informal coercive interventions in psychiatric emergencies [17]	Questionnaire with simulated cases to staff at one adult psychiatric hospital (*n* = 267)	(i) Informal interventions are preferred by staff (ii) Such interventions are typically not recorded and their importance may therefore be overlooked

7	Staff's perceptions (2)	Attitudes to coercion at two Norwegian psychiatric units [18]	Questionnaire with simulated cases to staff at two adult psychiatric units (*n* = 180)	There was a limited degree of variance in staff's responses with respect to degree of restrictiveness The study supported the idea that a range of different interventions are used in emergency situations

8	Rates, characteristics (3)	The care conditions for especially dangerous psychotic patients [19]	Review of records at one institution (Reitgjerdet)	(i) In 1977, physical restraint was used 15 444 days (22.4% of all patient days) (ii) In 1980, it was used 566 patient days (iii) In the first third of 1981, it was used only 1,5 patient days

9	Rates, characteristics (3)	Use of coercive measures in Norwegian psychiatric institutions [20]	Examination of records of mechanical restraint (“screening”)	During the first six months of 1988, 203 patients had been mechanically restrained for 10,767 hours

10	Rates, characteristics (3)	Changes in the use of coercive measures in Norwegian psychiatric institutions [21]	Examination of records of mechanical restraint (“screening”)	(i) 9402 hours of mechanical restraint were recorded in the first half of 1990 (ii) Single patients create large variations in use (iii) No correlation between size of ward or staff ratio and use of coercion (iv) No difference in levels from previous study (1986–1988)

11	Rates, characteristics (3)	Psychiatric security units in Norway; patients and activity [22]	Review of medical records of patients in Norwegian security units (*n* = 123)	25% had been subjected to physical restraint during a six-month period in 1993

12	Rates, characteristics (3)	Polar day and polar night: month of year and time of day and the use of physical and pharmacological restraint in a north Norwegian university psychiatric hospital [23]	Review of protocols and medical records regarding restraint during a 5.5-year period	(i) There was a daily peak with most use of restraint in the afternoon and early evening and a seasonal peak—with the most use of restraint in autumn (ii) Patterns of use might be caused by light-dark cycles, variations in life-events, and variations in the ward environment

13	Rates, characteristics (3)	Medicate, restrain, or seclude? Strategies for dealing with violent and threatening behaviour in a Norwegian university psychiatric hospital [24]	Review of protocols and medical records from a 5.5-year period at a psychiatric hospital	(i) 797 episodes of physical restraint were identified (ii) Patients' sex, age group, and diagnostic group were of importance to use (more use with male, younger, and nonpsychotic patients)

14	Rates, characteristics (3)	Association between patients' gender, age, and immigrant background and use of restraint—a 2-year retrospective study at a department of emergency psychiatry [25]	The study retrospectively examined routinely collected data and data from restraint protocols in a department of acute psychiatry over a 2-year period	(i) 14% of the patients were subjected to physical and/or pharmacological restraint (ii) The rate was significantly higher among patients with immigrant background, especially in the younger age groups

15	Rates, characteristics (3)	Staff injuries after patient-staff incidences in psychiatric acute wards [26]	507 patient-staff incidents in a psychiatric acute ward	Holding the patient with force was more frequent in incidents where more than one body part was injured

16	Rates, characteristics (3)	A cross-sectional prospective study of seclusion, restraint, and involuntary medication in acute psychiatric wards [9]	Medical records data from 1014 involuntarily admitted patients in acute psychiatric wards, of which 117 had been physically restrained	(i) Increased risk of restraint for patients that were aggressive or had tendency to self-injury (ii) Lower risk for patients from other ethnic groups (iii) Increased risk in urban areas

17	Rates, characteristics (3)	Characteristics of psychiatric inpatients who experienced restraint and those who did not: a case-control study [27]	Retrospective case-control study of records' data from three acute wards, two-year sample	Restrained patients (mechanically and pharmacologically) were more likely to be male, reside outside catchment area, have immigrant background, have longer stays, be involuntarily admitted, and have specific diagnoses (substance use, schizophrenia, psychoses, and bipolar disorder)

18	Rates, characteristics (3)	Mechanical and pharmacological restraints in acute psychiatric wards—why and how are they used? [8]	Data from records of patients (*n* = 306) that had been subjected to mechanical restraint in three acute wards during a two-year period	(i) Occurring or imminent assault was most frequent reason for restraint (ii) Diagnoses, age, and reason for restraint increased the likelihood of being subjected to specific types of restraint

19	Rates, characteristics (3)	Characteristics of patients frequently subjected to pharmacological and mechanical restraint—a register study in three Norwegian acute psychiatric wards [28]	Retrospective study of records' data from three acute wards	9.1% of those restrained (either mechanically or pharmacologically) had been so 6 or more times, accounting for 39.2% of all restraint episodes

20	Rates, characteristics (3)	Mechanical restraint in psychiatry: preventive factors in theory and practice [29]	Different data sources, including questionnaire to clinical nurse managers in Norway (*n* = 47)	Mandatory review, patient involvement, and no crowding were identified as preventive factors

21	Rates, characteristics (3)	Comparing the effect of nonmedical mechanical restraint preventive factors between psychiatric units in Denmark and Norway [30]	Cross-sectional survey of psychiatric units	The following factors were found to partly explain differences in restraint levels: staff education, substitute staff, acceptable work environment, separation of acutely disturbed patients, patient-staff ratio, and identification of the patients' crises triggers

22	Intervention studies (5)	Patients' perception of coercion in acute psychiatric wards: an intervention study [31]	Three interventions were used: patients' engagement in the formulation of treatment plans, patient and staff evaluations, and renegotiations of treatment plans. Data were obtained on self-rating scales	(i) The interventions resulted in marginal changes (in the staff's respect and understanding and total satisfaction) (ii) Actions taken to control behaviour (i.e., seclusion) were more strongly related to perceived coercion than aspects of compulsory treatment

23	Case studies (6)	Venous thromboembolism in connection with physical restraint [32]	Case report	Venous thromboembolism may occur in connection with physical restraint

24	Case studies (6)	Thrombosis associated with physical restraints [33]	Literature review and two cases	Immobilization and trauma to the legs while restraining a patient may lead to thrombosis

25	Case studies (6)	Physical restraint and near death of a psychiatric patient [34]	Literature review and case report	Physically restraining (holding) a patient in the prone position with a significant weight load on the torso can lead to asphyxiation

26	Ethnographic studies (7)	Creating trust in an acute psychiatric ward [35]	Ethnographic study (observation and interviews with five patients and six nurses) of an open seclusion unit	Distrust is prevalent, but trust can be created Regarding themselves as potential causes of distrust can contribute to nurses' developing a realistic view of their practice

27	Ethnographic studies (7)	Coercion in a locked psychiatric ward: perspectives of patients and staff [36]	Ethnographic study	Description of one patient's negative perceptions of physical restraint

28	Ethnographic studies (7)	Fear, danger, and aggression in a Norwegian locked psychiatric ward [37]	Ethnographic study	Physical restraint seen as a threat and means of control and as a reminder of danger
